# Investigation of Pain Mechanisms by Calcium Imaging Approaches

**DOI:** 10.1007/s12264-017-0139-9

**Published:** 2017-05-13

**Authors:** Michael Anderson, Qin Zheng, Xinzhong Dong

**Affiliations:** 1 The Solomon H. Snyder Department of Neuroscience, Center for Sensory Biology, School of Medicine, Johns Hopkins University, Baltimore, MD 21205 USA; 2 Howard Hughes Medical Institute, School of Medicine, Johns Hopkins University, Baltimore, MD 21205 USA

**Keywords:** DRG, Spinal cord, GCaMP imaging, Pain pathways, Neural circuit

## Abstract

Due to the complex circuitry and plethora of cell types involved in somatosensation, it is becoming increasingly important to be able to observe cellular activity at the population level. In addition, since cells rely on an intricate variety of extracellular factors, it is important to strive to maintain the physiological environment. Many electrophysiological techniques require the implementation of artificially-produced physiological environments and it can be difficult to assess the activity of many cells simultaneously. Moreover, imaging Ca^2+^ transients using Ca^2+^-sensitive dyes often requires *in vitro* preparations or *in vivo* injections, which can lead to variable expression levels. With the development of more sensitive genetically-encoded Ca^2+^ indicators (GECIs) it is now possible to observe changes in Ca^2+^ transients in large populations of cells at the same time. Recently, groups have used a GECI called GCaMP to address fundamental questions in somatosensation. Researchers can now induce GCaMP expression in the mouse genome using viral or gene knock-in approaches and observe the activity of populations of cells in the pain pathway such as dorsal root ganglia (DRG), spinal neurons, or glia. This approach can be used *in vivo* and thus maintains the organism’s biological integrity. The implementation of GCaMP imaging has led to many advances in our understanding of somatosensation. Here, we review the current findings in pain research using GCaMP imaging as well as discussing potential methodological considerations.

## Introduction

The sensation of pain involves interactions between many highly-specialized cell types in the peripheral and central nervous systems. Numerous studies have used electrophysiological recordings to better understand how the nervous system transmits painful information. Electrophysiology has been the gold standard for studying the neuronal components of pain sensation due to its exquisite temporal resolution and sensitivity. Electrophysiology is not without its challenges however. Successful recordings demand physical contact with the tissue, which necessitates many recordings to be taken from *ex vivo* or *in vitro* preparations. Intracellular recordings require penetration of the neuron with an electrode, which can cause neurons to lose biological integrity. In addition, *ex vivo* preparations require excision of the tissue from the organism, which may induce the release of many factors, further affecting the results. This pseudo-physiological environment may not fully reflect the cellular landscape. *In vivo* electrophysiological models have been produced to provide more accurate information from living animals, yet it is impossible to record the activity of large populations of neurons simultaneously.

**Fig. 1:**
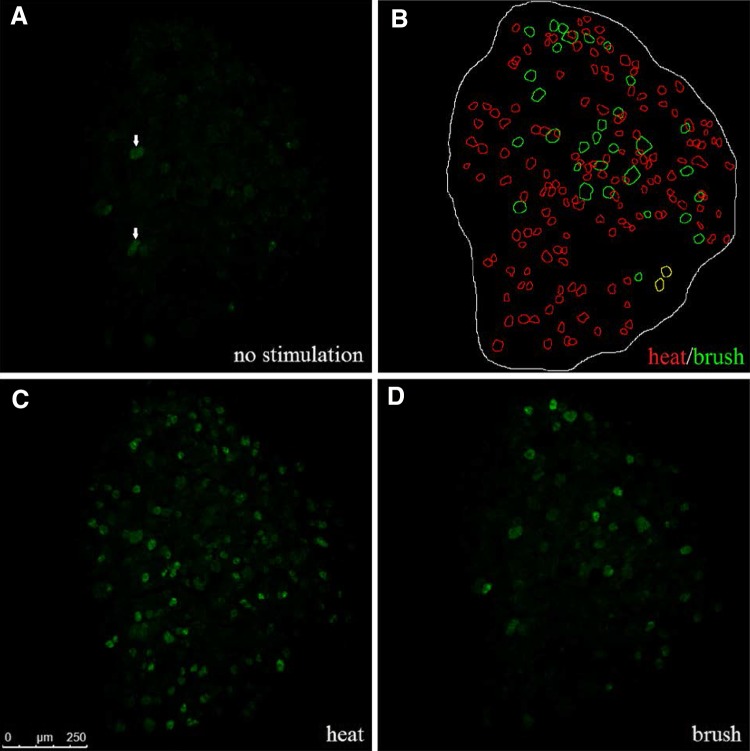
Representative *in vivo* DRG images from a *Pirt-cre;Rosa26-flox-stop-flox-GCaMP6s* heterozygous mouse. **A** Background GCaMP fluorescence in the absence of stimulation to the hind paw. *Arrows* indicate spontaneously firing neurons. **B** ROIs manually traced for neurons from panels **C** and **D** responding to noxious heat (*red*), brush (*green*), or both heat and brush (*yellow*). **C** Representative Ca^2+^ transient in response to placing the ipsilateral hindpaw in a beaker of 48 °C water. Cell diameters of responding neurons range from 11.3 to 33.1 μm. Mean cell diameter = 21.0 ± 0.3 μm. **D** Representative Ca^2+^ response to applying gentle brushing to the dorsal aspect of the ipsilateral hind paw. Cell diameters of responding neurons range from 18.7 to 44.6 μm. Mean cell diameter = 29.8 ± 1.1 μm.

Calcium imaging techniques using GECIs have been developed to image intracellular Ca^2+^ as an indirect measure of action potential firing [1]. GECIs have been used to study pain pathways by visualizing Ca^2+^ transients in response to various stimuli [2]. These methods use the less invasive properties of light rather than electrodes. Recently, an optical imaging technique has been developed using the GECIs GCaMP3 and GCaMP6s genetically expressed in primary sensory neurons under the control of the *pirt* promoter [3]. This technique allows for high-throughput, long-term, *in vivo* imaging of Ca^2+^ transients in primary afferents [4–6] (Fig. 1). Used in conjunction with genetic or pharmacological manipulations, this technique can provide extensive information on how painful stimuli are transmitted or altered in different pain states. In this review we summarize the current findings in pain research using GCaMP3 and GCaMP6 mice.

## Biochemistry of GCaMPs

GCaMPs are a series of GECIs consisting of a Ca^2+^-binding domain fused to one circularly permuted fluorescent protein (FP). The Ca^2+^-binding domain, calmodulin (CaM), is fused to the C-terminal of the FP, and the Ca^2+^/CaM-binding myosin light chain kinase domain (M13) is fused to the N-terminal of the FP. When Ca^2+^ is absent, the FP is in a poorly fluorescent state. CaM can bind up to four Ca^2+^ ions through E-F motifs. After Ca^2+^ binds to CaM it undergoes a conformational change and binds to the M13 domain *via* the hinge region. The interaction of the Ca^2+^-CaM and M13 domains enables de-protonation of the FP chromophore and induces vigorous excitation and emission [7].

Currently, GCaMPs are widely used to image various biological model systems including *Caenorhabditis elegans*, *Drosophila*, zebrafish, rodents, and non-human primates. The first generation of GCaMP (GCaMP1) was developed by Nakai and colleagues [8] and later improved upon by Ohkura *et al.* with the development of GCaMP1.6 [9]. All of the first-generation GCaMPs are unstable at temperatures over 30 °C so they cannot be used for tests in mammalian systems. To resolve this issue, Tallini *et al.* developed GCaMP2 and were the first to record mammalian GCaMP *in vivo* [10]. Recently, several GCaMP proteins such as GCaMP3 [11], GCaMP-HS, GCaMP5 [12], GCaMP6, GCaMP7 [13], and GCaMP8 [14] have been engineered to have better sensitivity, much greater signal-to-noise ratios, and different spectral properties. It is difficult to use GCaMPs in transgenic animals that already express other GFP-based proteins, so red fluorescent GECIs, such as the red-shifted variant R-GECO based on mApple and RCaMP based on mRuby, have been developed for expanding the application of multichannel imaging [15, 16].

## Imaging Peripheral GCaMP

Soon after the development of functional imaging in the brain, the application of functional Ca^2+^ imaging in the peripheral nervous system was successful. In one previous study, our lab performed *in vitro* functional Ca^2+^ imaging of the trigeminal system, including the peripheral terminals of the ear skin, central terminals in the trigeminal subnucleus caudalis, and the cell bodies in the trigeminal ganglion, with Pirt-GCaMP3 mice [3]. The advantages of imaging in that study are manifold, such as high-efficiency simultaneous imaging of multiple neurons and preservation of somatotopic organization. Compared to *in vitro* imaging, *in vivo* imaging can be used to study an intact animal and is more representative of the physiological condition. A recent study used Ca^2+^ imaging to record the responses of the central terminals of primary sensory neurons in the DRG to cutaneous stimulation *in vivo* [2]. The imaging of nerve terminals requires more stability because the diameters of the central terminals are very small and more sensitive to movement. The cell bodies of primary sensory neurons are much larger than terminals and are therefore less affected by micromovements. However, DRGs are located underneath the vertebrae and are surrounded by connective tissue and muscles which make them more difficult to image. That is one of the reasons why most studies on the function of DRGs in somatosensation have focused on the activity of one neuron in culture or populations of neurons in isolated ganglia.

With the development of a novel *in vivo* DRG imaging technique, researchers now have the ability to detect the activity of large populations of DRG neurons in anesthetized mice [4]. Using this technique, we can simultaneously monitor Ca^2+^ transients in large populations of DRG neurons (>1600 neurons/DRG, ~15% of total DRG neurons) in live Pirt-GCaMP3 mice. In addition, this new *in vivo* DRG imaging approach enables us to study large numbers of neurons under physiological conditions without cell penetration or dissociation. We found that, after injury, gap junction-mediated neuron-to-neuron coupling occurs in the DRG. This coupling phenomenon occurs between cells with varying somal diameters, including large-diameter neurons recruiting adjacent small-diameter neurons. Therefore, injury could lead to non-nociceptive stimuli activating innocuous primary afferent neurons, in addition to adjacent coupled nociceptors, potentially contributing to mechanical allodynia. Although neuronal “cross-activation” had been reported previously using dual recordings in the rat DRG [17, 18], our imaging study was the first to directly demonstrate neuronal coupling of neighboring neurons after injury. This imaging technique opens up new avenues for the study of the role of primary sensory neurons in different research fields. More recently, another study used this technique to show that >85% of responsive DRG neurons are modality-specific, responding to either noxious mechanical, cold, or heat stimuli [6]. This result contrasts with past findings using electrophysiological recordings which indicated that most DRG neurons respond to more than one modality. Although follow-up studies are necessary to explore the factors involved in C-fiber polymodality, this study provides some interesting arguments when considering the common view that most C-fibers are polymodal in naïve animals.

Another study using GCaMP expressed in all neurons reports that inflammation can cause a change in the number of DRG neurons that are responsive to mechanical stimulation [19]. Interestingly, they show that some DRG neurons exhibit no change in Ca^2+^, yet these neurons can generate action potentials. They go on to show that the neurons that have no change in Ca^2+^ show narrow action potentials and rapidly-conducting axons. A separate study monitored the response of primary sensory neurons to different temperatures using *in vivo* imaging of the trigeminal ganglion, and revealed that TRPV1 channels are required for warm sensation and one class of silent cold sensors emerges after injury [20]. One similarity between all of these studies that have used GCaMP imaging of live animals is that they keep the biological integrity of the primary sensory neuron system intact while simultaneously monitoring the activity of a large number of neurons.

## Imaging Spinal GCaMP

The development of two-photon imaging has allowed for the visualization of fluorescent targets deep within tissue [21]. In the past decade, groups have used this imaging technique to visualize neurons and glia in the spinal cord [22, 23]. Two-photon imaging, coupled with Ca^2+^-sensitive dyes, has allowed continuous imaging of cellular activity in the spinal cord [24, 25]. This technique can yield crucial information on how spinal neurons and glia modulate somatosensory transmission and elucidate regulatory circuits in the spinal cord.

Recently, Sekiguchi *et al.* combined one- and two-photon microscopy and GCaMP imaging to visualize neuronal and glial activity in freely-moving unanesthetized mice while applying different stimuli to the base of the tail [26]. To induce GCaMP6f expression in the spinal cord, they injected either AAV9-CaMKII-GCaMP6f (for neurons) or AAV5-GfaABC1D-GCaMP6f (for glia) into the spinal cord and subsequently imaged the Ca^2+^ transients using two-photon and miniaturized one-photon microscopy. They observed an intensity-dependent Ca^2+^ response at the single-neuron level. In addition, astrocytes exhibited a coordinated Ca^2+^ response to high-amplitude pinch stimulation. The use of GCaMP6 expressed in neurons or glia allowed the simultaneous imaging of many cells, which would not be possible using current electrophysiological techniques.

## Methodology

When using GCaMP for *in vivo* imaging, there are many technical concerns to consider. Different microscopic avenues offer distinct advantages and disadvantages. Two-photon microscopy may be necessary when imaging targets lie deep within a specimen. Additional adaptations such as adaptive optics, highly sensitive detectors such as GaAsP detectors, or objectives with higher numerical apertures may assist in imaging targets with low fluorescence. When using two-photon imaging, stabilization of the target tissue is paramount. Fortunately, there are ways to circumvent this issue, such as implantable spinal windows [22, 26], vertebral clamps [4, 6, 23, 24], a thin layer of agarose over the tissue, or an adaptive focus control unit [25, 26]. One-photon microscopy is less sensitive to movement artifacts in the z-direction due to its higher point spread function; however, tissue-wide delivery of high-intensity laser light introduces the concern of phototoxicity out of the imaging plane. In addition, it can be challenging to use one-photon imaging in highly-scattering tissue such as the spinal cord.

It is also very important to address variable expression levels of GCaMP between neurons. The level of GCaMP can directly affect the level of fluorescence observed upon activation. Insertion of GCaMP into the mouse genome using a viral vector or gene knock-in approach can result in variable GCaMP expression between neurons. This factor can make it difficult to compare Ca^2+^ transients between different cell types. Consequently, when choosing an appropriate strategy for GCaMP insertion into the mouse genome it is imperative that one should take the expression levels into consideration and interpret the results accordingly.

## Limitations of Imaging

Imaging enables minimally-invasive observation of neural activity in real time. It is an ideal and powerful approach to simultaneously monitor the activity of large numbers of neurons in live animals. While imaging approaches have improved with the development of fluorophore sensors and microscopic systems, it is important to consider the potential limitations (Table 1).

**Table 1:** Advantages and disadvantages of* in vivo* GCaMP imaging.

Advantages	Disadvantages
Simultaneous visualization of Ca^2+^ transients in large populations of cells	Can be difficult to observe targets in highly scattering tissue
Excellent spatial resolution	Limited temporal resolution
Improved preservation of physiological condition	Not a direct measurement of action potential firing
Does not require application or injection of Ca^2+^-sensitive dyes	Can be susceptible to effects of Ca^2+^-buffering
Uses minimally-invasive properties of light	Cannot measure neurophysiological characteristics such as action potential number, frequency, duration
Maintenance of somatotopic organization	Level of fluorescence can be affected by variable GCaMP expression levels
Can be inserted into mouse genome, allowing for easy and repeatable expression between generations	Susceptible to effects of phototoxicity
Can be combined with other fluorophores for multichannel imaging	

First, imaging of Ca^2+^ influx is an indirect measurement of neural activity, as Ca^2+^ plays many roles other than neuronal excitation. In fact, it has been reported that in some cases the activity of neurons is not necessarily associated with a change in Ca^2+^ concentration [27]. Second, it is impossible to deduce several of the characteristics of neuronal excitation such as action potential duration, number, and frequency, as well as other intrinsic electrophysiological properties of responding neurons using Ca^2+^ imaging. Third, the temporal resolution of GCaMP imaging remains a recurring issue. The scanning speed used to record the Ca^2+^ dynamics is in the order of seconds to hundreds of milliseconds, yet action potentials are generated at much faster rates. Therefore, there is the potential for lost information without performing parallel electrophysiological recordings. Fourth, when using anesthesia it is important to understand the physiological impact on the tissue. Although the mechanisms of action are unclear for many anesthetics it is thought that some, such as isoflurane, work through facilitating inhibitory transmission or inhibiting excitatory transmission [28]. In fact, a recent study shows that isoflurane potently suppresses Ca^2+^ activity in neurons and glia in the spinal dorsal horn [26]. Therefore, care should be taken to use proper controls or confirm the phenotype under different anesthetic conditions. Fifth, since GCaMP binds to intracellular Ca^2+^, it can alter the concentration of free Ca^2+^ ions. This Ca^2+^-buffering effect can lead to robust cellular changes, which can ultimately affect cellular vitality. In fact, high GCaMP expression levels introduced using viral vectors have been associated with abnormal cellular physiology [29]. The Ca^2+^-buffering effect could be further intensified in cells with high levels of endogenous Ca^2+^-binding proteins such as Purkinje cells [30]. However, a cytotoxic effect of GCaMP has not been found in DRG neurons, especially in Pirt-GCaMP mice [3, 4]. Finally, even with the recent advances in multiphoton imaging, it can still be difficult to achieve the desired imaging depth in highly myelinated tissue such as the spinal cord. New developments may be needed to allow the imaging of deeper targets.

## Future Perspectives

This new imaging technique can be of great value for studying neurons and non-neuronal cells. In addition to neurons, several non-neuronal cells such as keratinocytes, immune cells, glia, and cancer cells are involved in the pathogenesis of pain. GCaMP imaging can be combined with optogenetic methods to study the interactions of neuronal and non-neuronal cells *in vivo.* This may prove to be a reliable tool to study microcircuits and neuron-neuron and neuron-glia interactions. In addition, red-shifted Ca^2+^ indicators such as RCaMP can be combined with GCaMP to allow the imaging of several sub-populations of cells simultaneously.
